# Tumour suppressor EP300, a modulator of paclitaxel resistance and stemness, is downregulated in metaplastic breast cancer

**DOI:** 10.1007/s10549-017-4202-z

**Published:** 2017-03-24

**Authors:** Muhammad Asaduzzaman, Stephanie Constantinou, Haoxiang Min, John Gallon, Meng-Lay Lin, Poonam Singh, Selina Raguz, Simak Ali, Sami Shousha, R. Charles Coombes, Eric W.-F. Lam, Yunhui Hu, Ernesto Yagüe

**Affiliations:** 1 Division of Cancer, Imperial College Faculty of Medicine, Hammersmith Hospital Campus, Du Cane Road, London, W12 0NN UK; 2 Centre for Pathology, Department of Medicine, Imperial College Faculty of Medicine, Charing Cross Hospital, Fulham Palace Rd, London, W6 8RF UK; 3 Division of Clinical Sciences, Imperial College Faculty of Medicine, Hammersmith Hospital Campus, Du Cane Road, London, W12 0NN UK; 4 The 3rd Department of Breast Cancer, China Tianjin Breast Cancer Prevention, Treatment and Research Center, National Clinical Research Center of Cancer, Tianjin Medical University Cancer Institute and Hospital, Huan Hu Xi Road, Ti Yuan Bei, He xi District, Tianjin, 300060 People’s Republic of China; 5 Present Address: Department of Clinical Pharmacy and Pharmacology, University of Dhaka, Dhaka, 1000 Bangladesh; 6 Present Address: MRC Cancer Unit, Hutchison/MRC Research Centre, Cambridge, CB2 0XZ UK

**Keywords:** Metastasis, Cancer stem cells, Drug resistance, BCL2, ABCG2, EP300 signature

## Abstract

**Purpose:**

We have previously described a novel pathway controlling drug resistance, epithelial-to-mesenchymal transition (EMT) and stemness in breast cancer cells. Upstream in the pathway, three miRs (miR-106b, miR-93 and miR-25) target EP300, a transcriptional activator of E-cadherin. Upregulation of these miRs leads to the downregulation of EP300 and E-cadherin with initiation of an EMT. However, miRs regulate the expression of many genes, and the contribution to EMT by miR targets other than EP300 cannot be ruled out.

**Methods:**

We used lentiviruses expressing EP300-targeting shRNA to downregulate its expression in MCF-7 cells as well as an *EP300*-knocked-out colon carcinoma cell line. An EP300-expression plasmid was used to upregulate its expression in basal-like CAL51 and MDA-MB-231 breast cancer cells. Drug resistance was determined by short-term proliferation and long-term colony formation assays. Stemness was determined by tumour sphere formation in both soft agar and liquid cultures as well as by the expression of CD44/CD24/ALDH markers. Gene expression microarray analysis was performed in MCF-7 cells lacking EP300. EP300 expression was analysed by immunohistochemistry in 17 samples of metaplastic breast cancer.

**Results:**

Cells lacking EP300 became more resistant to paclitaxel whereas EP300 overexpression increased their sensitivity to the drug. Expression of cancer stem cell markers, as well as tumour sphere formation, was also increased in EP300-depleted cells, and was diminished in EP300-overexpressing cells. The EP300-regulated gene signature highlighted genes associated with adhesion (*CEACAM5*), cytoskeletal remodelling (*CAPN9*), stemness (*ABCG2*), apoptosis (*BCL2*) and metastasis (*TGFB2*). Some genes in this signature were also validated in a previously generated EP300-depleted model of breast cancer using minimally transformed mammary epithelial cells. Importantly, two key genes in apoptosis and stemness, *BCL2* and *ABCG2*, were also upregulated in EP300-knockout colon carcinoma cells and their paclitaxel-resistant derivatives. Immunohistochemical analysis demonstrated that EP300 expression was low in metaplastic breast cancer, a rare, but aggressive form of the disease with poor prognosis that is characterized by morphological and physiological features of EMT.

**Conclusions:**

EP300 plays a major role in the reprogramming events, leading to a more malignant phenotype with the acquisition of drug resistance and cell plasticity, a characteristic of metaplastic breast cancer.

**Electronic supplementary material:**

The online version of this article (doi:10.1007/s10549-017-4202-z) contains supplementary material, which is available to authorized users.

## Introduction

Many breast cancers initially respond well to chemotherapy, however, in a significant proportion of cases, and following a cancer-free period, metastases at distant sites appear and these are accompanied by a poor response to therapy, normally with fatal consequences. It is thus imperative to improve our understanding of the mechanisms leading to cancer spread and therapy response with the aim of developing new markers and therapeutic strategies [1].

Epithelial cells undergoing epithelial-to-mesenchymal transition (EMT, the first step in the metastatic cascade) lose E-cadherin expression and their characteristic epithelial morphology. These events are accompanied by an increase in motility and acquisition of the ability to degrade the extracellular matrix and invade other tissues. Loss of E-cadherin, a hallmark of the EMT programme, liberates β-catenin (normally associated with the C-terminus of E-cadherin), which migrates to the nucleus and induces the expression of genes orchestrating the EMT programme. Importantly, the EMT programme generates cancer cells with stem cell-like characteristics (cancer stem cells, CSCs). EMT and CSCs are often associated with the acquisition of drug resistance. Thus, these three processes are tightly interlinked [2–4].

Downregulation of E-cadherin by transcriptional repressors (Snail and Slug, as well as ZEB1, ZEB2 and Twist) is well-studied. However, transcriptional activators of E-cadherin, which help maintain the cell in an epithelial state, such as EP300, FOXA1 and RUNX1, are less well characterized [5]. EP300 was originally discovered as a transcriptional co-activator that plays pivotal roles in integrating and mediating multiple signal-dependant transcription events [6]. The most studied function of EP300 is as a histone acetyltransferase, regulating transcription via chromatin remodelling [7], and it has important roles in cell proliferation, transformation and differentiation [8]. Loss of EP300 heterozygosity has been described in breast carcinomas [9], somatic *EP300* mutations have been identified in several malignancies [10] and EP300-deficient colon carcinoma cells show phenotypic changes characteristic of EMT [11].

We have recently described a novel EMT/CSC/drug resistance regulatory axis controlled by the miR-106b~25 cluster [12], which is associated with aggressive basal-like, oestrogen receptor-negative, grade 3 breast cancers [13]. The three miRs in the cluster target *EP300* mRNA and downregulate its expression, leading to increased motility and invasive properties as well as the generation of doxorubicin and radiation-resistant derivatives [12]. In bladder cancer cells, experimental downregulation of EP300 also leads to doxorubicin and cisplatin resistance [14], [15]. Using a minimally transformed mammary epithelial cell model [16], we have also demonstrated that cells in which this pathway has been experimentally downregulated acquire a multidrug resistance phenotype with evasion from apoptosis [17].

Here we show that experimental modulation of EP300 alters paclitaxel sensitivity and the generation of paclitaxel resistance. EP300 silencing is also associated with increased in vitro tumorigenicity and CSC-like markers, whilst its ectopic expression in basal-like breast cancer cells partly rescues the epithelial, differentiated and paclitaxel-sensitive phenotype. Gene expression profiling identifies down-stream EP300 targets associated with drug resistance, EMT and CSCs. Finally, immunohistochemical analysis reveals a strong downregulation of EP300 in metaplastic breast cancer, a rare, but aggressive form of invasive breast cancer with histological evidence of EMT, which has a poor clinical outcome.

## Materials and methods

### Cells

MCF-7 and MDA-MB-231 cells were obtained from Sigma-Aldrich, CAL51 cells from the German Resource Centre for Biological Material (DSMZ), HCT116 and HCT-KOEP300 (a genetic EP300 knockout from HCT116 cells [11]) cells from Cancer Research Technology and HEK293T cells from the American Type Culture Collection. Minimally transformed human mammary epithelial cells (MTMEC) were a gift from William Hahn (Dana Farber, Boston). MTMECs express TERT, SV40 large T antigen, a constitutively active form of PI3K, p110α and oncogenic ras [16] and were maintained in serum-free HuMEC medium (Life Technologies). HEK293T were maintained in DMEM supplemented with 4.5 g/L glucose, 10% foetal calf serum and 4 mM l-glutamine (Life Technologies). MCF-7 and MDA-MB-231 cells were maintained as HEK293T cells but with 1 g/L glucose. HCT116 cells were maintained in McCoy’s 5A medium supplemented with 10% foetal calf serum and 4 mM l-glutamine.

Downregulation of *EP300* was obtained by stable expression of hairpins in the lentiviral vector pGIPZ^®^ (Thermo Scientific). Two different hairpins,

V3LHS_331296 (mature antisense: TGTGCACAACTGTTTGCCG) and V3LHS_331295 (mature antisense: TAATCTATCTTCAGTAGCT), from the RNAi Consortium (Broad Institute) were used. Viral transductions were essentially as described [18] and cells were selected and maintained with 1 µg/mL puromycin. Overexpression of *EP300* was obtained by stable expression of a pcDNA3.1-derived construct carrying the full-length *EP300* cDNA (Addgene #23252) [19]. Cells were transfected with GenJet (SignaGen Laboratories) following manufacturer’s instructions and selected and maintained with 1 mg/mL G418. Pools of at least 200 G418-resistant clones were used in all cases. Paclitaxel-resistant lines were generated following a “pulse” methodology as described [12]. In short, cells were treated with paclitaxel (20 nM for MCF7-shEP300, 15 nM for MTMEC-shEP300 and 40 nM for HCT-KOEP300 derivatives) for 3 days, after which the cells were grown drug-free for two passages before repeating the drug treatment. Resistant lines were obtained after several months and did not show any significant cell death after drug treatment.

### Gene expression analysis

Total RNA (isolated using a RNeasy kit, Qiagen) was reverse transcribed with MuLV reverse transcriptase (High-Capacity RNA-to-cDNA kit, Applied Biosystems) and real-time quantitative PCR (QPCR) was performed using SYBR Green (Applied Biosystems) on an ABI Prism 7700 detection system (PerkinElmer Life Sciences). *RPS14* and *RPLP0* mRNAs were used as normalizers. A comparative threshold cycle was used to determine the relative gene expression as previously described [20]. Oligonucleotides used for gene expression analysis are shown in Supplementary Table 1.

### Antibodies

Antibodies for immunodetection following standard immunoblotting procedures were 24E10 for E-cadherin (Cell Signalling Technology), ab10485 for EP300 (Abcam), AC-15 (ab6276; Abcam) for β-actin and Bcl-2 (100) [sc-509] and C-5 (sc-365962) for Lamin B1 (Santa Cruz Biotechnology). Membranes were incubated with anti-rabbit (926-32213, IRDye^®^ 800CW Donkey anti-Rabbit, LI-COR) and anti-mouse (926-68072, IRDye^®^ 680RD Donkey anti-Mouse, LI-COR) secondary antibodies and the bands were visualised and quantified using an Odyssey Infrared Imaging System (LI-COR Biotechnology-UK Ltd).

### Drug sensitivity assay

The drug concentration necessary to kill 50% of cells (IC_50_) after 3 days of culture (6-well dishes; 3000 cells/well) was obtained after sulphorhodamine B (Sigma-Aldrich) staining [21] as previously described [22].

### Drug resistance clonogenic assay

Cells (2 × 10^5^) were seeded, at least in triplicate, in 25 cm^2^ culture flasks and exposed to a single dose of paclitaxel (Tocris Bioscience) for 3 days. Cells were kept in culture for 21 days with drug-free medium changes every three days. Drug-resistant clones were fixed with 4% paraformaldehyde and stained with 0.2% crystal violet and counted.

### Anchorage-independent growth assays

To assess the capacity of CSCs to propagate in an anchorage-independent manner, both soft agar and sphere assays were performed. Cells (5 × 10^4^) were seeded in 0.3% agar noble in complete DMEM medium on 30 mm plates with a bottom layer of solidified 0.6% agar noble in the same medium. Triplicate cultures for each cell type were maintained for 4 weeks at 37 °C in an atmosphere of 5% CO_2_ and 95% air, with 200 μL fresh medium added once a week. Colonies larger than 50 μm in diameter were counted after 4 weeks and stained with crystal violet. For the sphere formation assay [23], cells (100–200) were plated in each well of an ultralow attachment 24-well plate (Corning) with 3 mL serum-free mammary epithelial growth medium (MEGM, BioWhittaker), supplemented with 2% B27 supplement, 20 ng/mL epidermal growth factor and 20 ng/mL fibroblast growth factor-basic (all from Invitrogen). Cells were grown for 14 days, replenishing 500 µL of medium every 3–5 days, until spherical clusters larger than 50 µm diameter were counted.

### Flow cytometry

For stem cell markers, CD44 (G44-26, APC) and CD24 (ML5, PE), both from BD Biosciences, were used essentially as described [23]. An Aldefluor assay kit (StemCell Technologies) was used for the determination of aldehyde dehydrogenase (ALDH) activity by flow cytometry essentially as described [23]. Briefly, cells were resuspended in assay buffer (10^6^ cells/mL). Activated Aldefluor substrate (5 μL) was added to samples and incubated at 37 °C for 45 min to allow substrate conversion. A sample with the ALDH inhibitor diethylaminobenzaldehyde was used as a negative control.

### Microarray hybridization, processing and data analysis

Total RNA was prepared from three independent biological replicates of each cell line using a RNeasy Kit (Qiagen). RNA integrity was evaluated using an Agilent 2100 Bioanalyzer. cDNA synthesis and further processing was performed using GeneChip WT PLUS Reagent kit (Affymetrix). Hybridization to Affymetrix GeneChip Human Gene ST Arrays followed manufacturer’s protocol. The control probes used to measure microarray hybridization efficiency were obtained from the Affymetrix online database and were filtered out before analysis. Raw signal intensity, background correction, quantile normalization, log_2_ transformation and probeset summarization were performed with Affymetrix Expression Console Software. Differential gene expression was analysed using Partek Genomic Suite (Partek, USA). The microarray data were deposited with the NCBI Gene Expression Omnibus (http://ncbi.nlm.nih.gov/geo/) under accession number GSE76200.

### Immunohistochemistry

A total of 17 FFPE metaplastic breast cancer samples and 17 normal (reduction mammoplasty/benign) breast tissue samples were used. Samples were collected from the Imperial College Healthcare Tissue Bank (R14086) and Breast Cancer Now Tissue Bank (BCNTB-TR000054) after ethics approval by both institutions. Immunohistochemical staining was performed using antibodies against EP300 (HPA003128, Sigma-Aldrich, dilution 1:200) and E-cadherin (Clone 36, #610181, BD Biosciences, dilution 1:200). EP300 antibody specificity was validated with a synthetic peptide (APrEST73567, Atlas Antibodies) competition assay following manufacturer’s protocol. A standard operating procedure for immunohistochemical staining (Charing Cross Hospital Histopathology Laboratory, Q-Pulse index Code: CEL-LP-172-X, Ver. 1.6, 2013, Supplementary Table 2) modified to use the Bond Polymer Refine Detection Kit (DS9800, Leica Biosystems) was used. All sections were visualised with diaminobenzidine and counterstained with haematoxylin. EP300 and E-cadherin immunoreactivity was scored based on staining intensity ranging from 0 to +3: 0 = null, +1 = low, +2 = intermediate and +3 = high, as described [24]. More than six representative fields of each slide were analysed for determining EP300 and E-cadherin expression levels. The percentage of EP300- or E-cadherin-positive tumour cells scored as high (+2 or +3 in at least 66% of cells) or low/none was calculated for each slide by two investigators (MA and EY) and validated by a pathologist (SS) and clinical oncologist (RCC).

### Statistical analysis

Statistical evaluations were performed by Student’s *t* test for paired data or one-way ANOVA with Dunnett’s multiple comparison test. Data were considered significant at a *P*-value inferior to 0.05.

## Results

### Modulation of EP300 in cell model systems

**Fig. 1:**
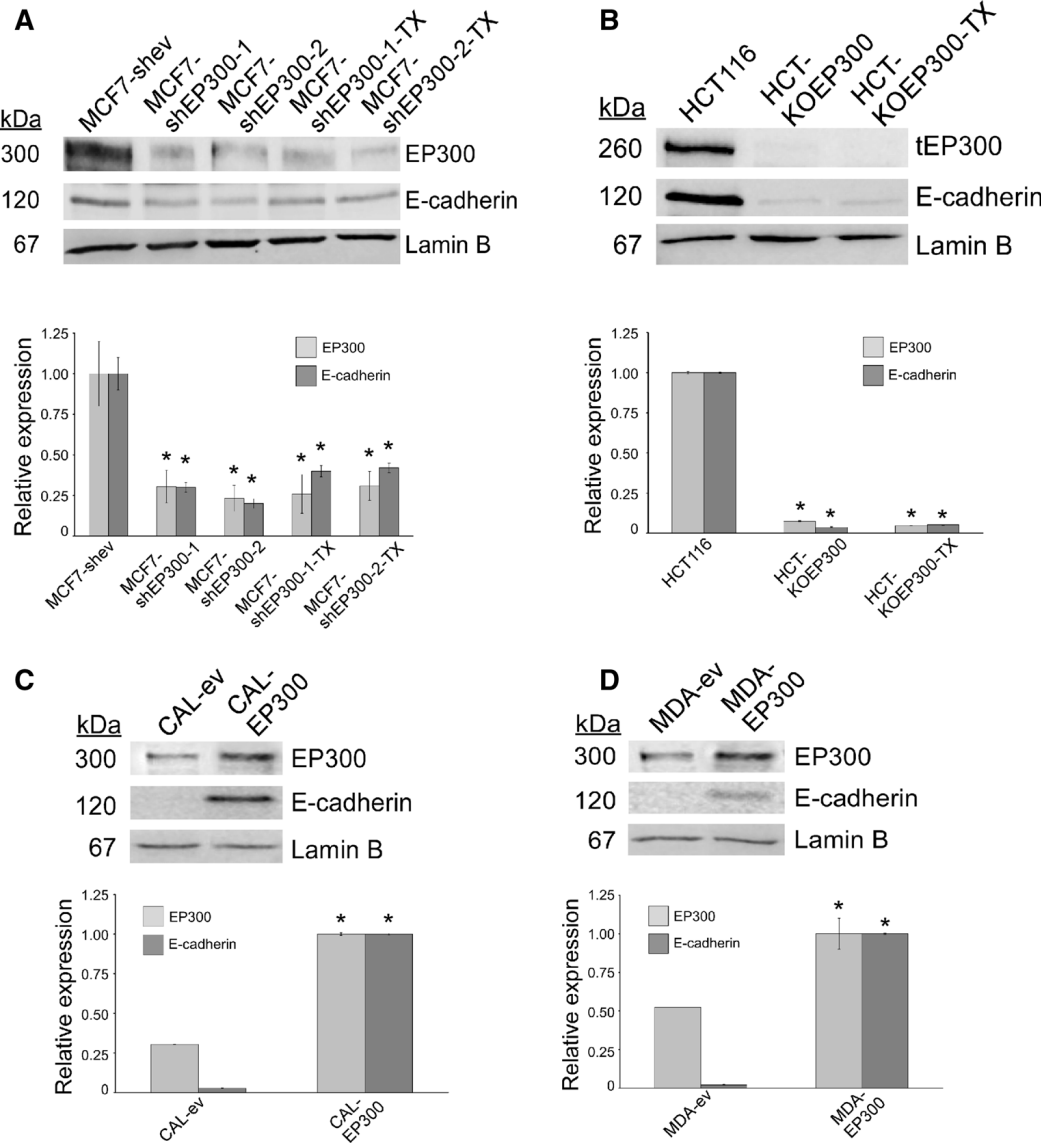
Experimental modulation of EP300 in cellular models. Expression of EP300 and E-cadherin was determined by immunoblot analyses. **a** EP300 was downregulated in breast cancer luminal MCF-7 cells by lentiviral expression of two different EP300 hairpins (MCF7-shEP300-1 and MCF7-shEP300-2). Cells expressing the empty pGIPZ vector (MCF7-shev) were used as control. **b**, a genetic knock-out of EP300 (HCT-KOEP300) is available in colon carcinoma HCT116 cells. This cell line is hemizygous for the *EP300* locus and generates a C-terminus truncated EP300 protein [10]—note its lower molecular mass (tEP300, truncated EP300). Paclitaxel-resistant derivatives are indicated with the -TX name extension. **c**, **d** EP300 was upregulated in breast cancer basal-like CAL51 and MDA-MB-231 cells with an EP300 expression cassette in pcDNA3.1 (CAL-EP300 and MDA-EP300). In both cases, cells transfected with pcDNA3.1 were used as controls (CAL-ev and MDA-ev). Lamin B was used as a loading control. Representative pictures of three replicates are shown. Immunoblots were quantified and data are shown in the histograms as average ± SD of three blots. All statistical comparisons (*P < 0.05) versus control cells

In order to study how breast cancer epithelial cells respond to EP300 downregulation, we generated stably transfected MCF-7 cells with a lentivirus driving the expression of hairpins targeting *EP300* mRNA. EP300 expression was high in this luminal cell line, but decreased to a high extent after the expression of two different EP300 hairpins. As expected, downregulation of EP300 led to a dramatic downregulation of E-cadherin (Fig. 1a).

HCT116 colon carcinoma cells are hemizygous for EP300 and due to the high frequency of homologous recombination in these cells, an EP300 genetic knocked-out model showing characteristics of EMT, has previously been generated [11]. These cells show, as expected, a lack of both EP300 and E-cadherin (Fig. 1a) and thus represent also a good cell system to study the phenotypic effects in an *EP300*-null background.

Triple-negative, basal-like breast cancer cells show many mesenchymal characteristics and thus we generated stably transfected cells derived from CAL51 and MDA-MB-231 in which EP300 was upregulated after transfection with an expression vector. Both CAL51 and MDA-MB-231 cells expressed low to moderate levels of EP300 that were upregulated in the stably overexpressed lines. As expected, E-cadherin was not detectable in the control cells, but its expression was upregulated in both CAL-EP300 and MDA-EP300 cells, although at a higher extent in the former than the latter (Fig. 1b).

### Modulation of EP300 expression alters paclitaxel sensitivity and the generation of paclitaxel-resistant cells

Paclitaxel is used both in the primary setting to reduce the risk of recurrence and in the advanced stage after lack of response to anthracyclines. We have previously shown that in a minimally transformed cellular model of breast cancer, experimental downregulation of EP300 decreases paclitaxel sensitivity and leads to the generation of paclitaxel-resistant cells [17]. In order to ascertain the generalized effect on paclitaxel response, we determined its IC_50_ values in the cell models generated above. EP300 downregulation led to an increase in the IC_50_ in MCF-7 cells, giving between a 3.8- and 5.7-fold increase in resistance depending on the hairpin used (Table 1). Importantly, this increase in paclitaxel resistance was also reproduced in EP300-knocked-out colon carcinoma HCT116 cells with an IC_50_ increase of 4.6-fold (Table 1). Conversely, when EP300 was experimentally upregulated in CAL51 and MDA-MB-231 cells, an increase in paclitaxel sensitivity (2.3- and 3.5-fold, respectively) was observed (Table 1).

**Table 1:** Modulation of paclitaxel sensitivity after experimental modulation of EP300 expression in cell models

Cell type	Cell derivative	EP300 status	IC_50_ (nM)^a^	Fold resistance	Fold sensitivity
MCF7	MCF7-shev		1.3		
	MCF7-shEP300-1	Downregulated	5.0	3.8	
	MCF7-shEP300-2	Downregulated	7.5	5.7	
	MCF7-shEP300-1-TX	Downregulated	45.6	35.1	
	MCF7-shEP300-2-TX	Downregulated	29.6	22.7	
HCT116	HCT116		3.1		
	HCT-KOEP300	Knockedout	14.2	4.6	
	HCT-KOEP300-TX	Knockedout	53.0	17.1	
CAL51	CAL-ev		4.2		
	CAL-EP300	Upregulated	1.8		2.3
MDA-MB-231	MDA-ev		32.1		
	MDA-EP300	Upregulated	9.2		3.5

^a^Paclitaxel concentration at which proliferation after 3 days was reduced by 50% estimated from the corresponding dose–response curves

**Fig. 2:**
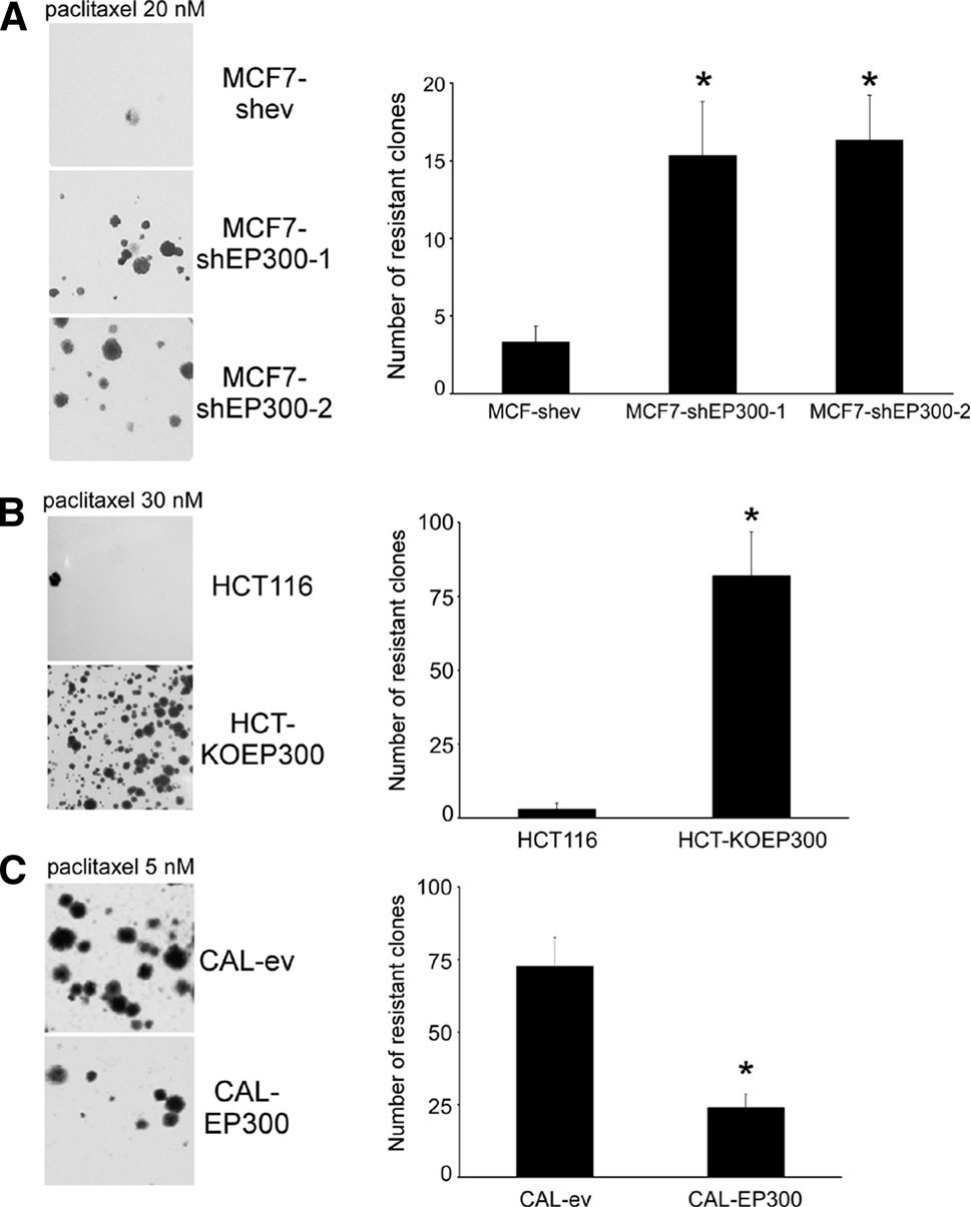
EP300 regulates the generation of paclitaxel resistance. Cells were treated for 3 days with paclitaxel and drug-resistant clones were stained with crystal violet 3 weeks after (*left panels*) and counted (*right panels*). **a**, MCF-7 cells. **b**, HCT116 cells. **c**, CAL51 cells. Numerical data represent the average ± SD of three independent experiments. All statistical comparisons (*P < 0.05) versus control cells. *Pictorial data* show a representative of three different experiments

Modulation of EP300 did not only affect short-term drug sensitivity, but also the long-term generation of drug-resistant cells. After 3 weeks of culture, and following an initial single paclitaxel treatment during 3 days, the number of drug-resistant clones increased approximately five-fold in MCF7-shEP300 (Fig. 2a) and 15-fold in HCT-KOEP300 cells (Fig. 2b). Conversely, overexpression of EP300 led to a reduction in the number of resistant clones by approximately three-fold in CAL-EP300 cells (Fig. 2c). When pools of resistant clones were isolated (MCF7-shEP300-1-TX, MCF7-shEP300-2-TX and HCT-KOEP300-TX), their paclitaxel IC_50_ values indicated a further decrease in drug sensitivity (Table 1). Thus, modulation of EP300 alters paclitaxel resistance.

### Modulation of EP300 expression alters cancer stem cell markers and anchorage independence

**Fig. 3:**
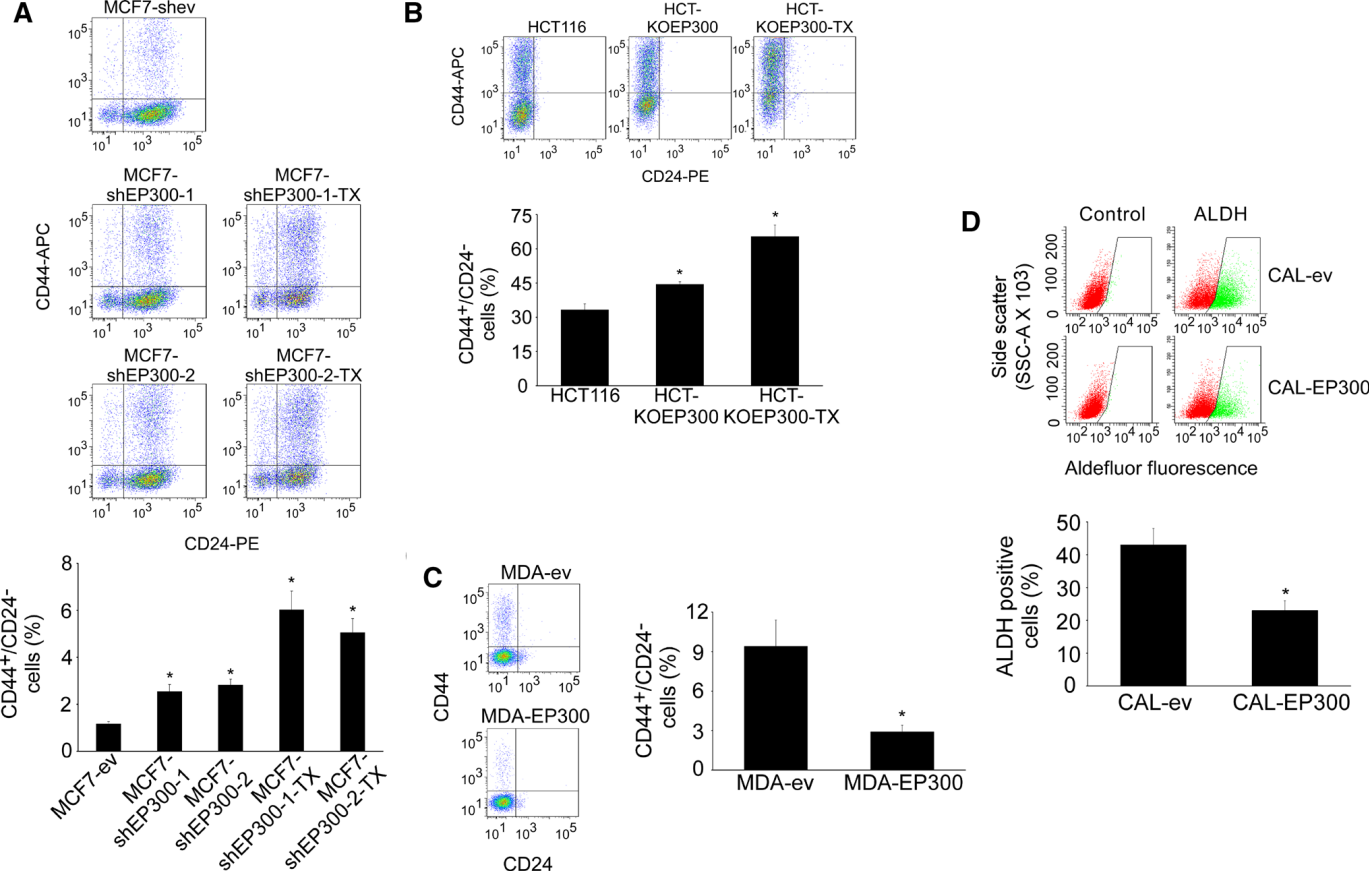
EP300 regulates stem cell markers. **a**–**c** Flow cytometry plots after staining (**a** MCF-7 cells; **b** HCT116 cells; **c** MDA-MB-231 cells) with CD44-APC- and CD24-PE-conjugated antibodies. Paclitaxel-resistant cell derivatives (MCF7-shEP300-1-TX, MCF7-shEP300-2-TX and HCT-KOEP300) were generated from the corresponding cells after selection with 20 nM (MCF-7 cells) and 40 nM paclitaxel (HCT116 cells). Histograms indicate the percentage of CD44^+^/CD24^−^ cells. **d** Upregulation of EP300 in CAL51 cells reduces the percentage of ALDH^+^ cells. Cells were treated with Aldefluor alone (*ALDH*) or in the presence of the ALDH inhibitor diethylaminobenzaldehyde (*Control*) and then analysed by flow cytometry. The green gate was set up with the control cells to include no more than 1% of the population and was used to determine the percentage of ALDH-positive cells in the absence of inhibitor. Representative flow cytometry plots are shown. Numerical data represent the average ± SD of three independent experiments. All statistical comparisons (*P < 0.05) versus control cells

Drug resistance is strongly associated with CSC characteristics, such as a CD44^+^/CD24^−^/ALDH1^+^ phenotype, and the ability to survive and grow in an anchorage-independent manner [25, 26]. We asked, first, whether modulation of EP300 would alter the proportion of CSCs. For this, we performed a double stain with CD44 and CD24 antibodies and determined the percentage of cells positive for each marker. As expected, MCF7-ev control cells showed a low proportion of CD44^+^/CD24^−^ cells (~1%). However, there was an increase in both MCF7-shEP300 (to 2.5–3%) as well as in their paclitaxel-resistant derivatives (to 5–6%; Fig. 3a). The increase in the CD44^+^/CD24^−^ population upon downregulation of EP300 was further confirmed using HCT116 cells. Although these cells have a high proportion of CD44^+^/CD24^−^ cells, EP300 knock-down led to a further increase in this subpopulation, from ~33% in HCT116 cells to ~44% in HCT-KOEP300 cells, that increased further to ~65% in HCT-KOEP300-TX-resistant cells (Fig. 3b). Conversely, experimental upregulation of EP300 expression led to a decrease in the CD44^+^/CD24^−^ subpopulation of MDA-MB-231 cells, from ~9% in MDA-ev to ~3% in MDA-EP300 cells (Fig. 3c). Despite its triple-negative and basal characteristics, CAL51 cells have a very low CD44 expression [27]. However, determination of ALDH^+^ cells by flow cytometry confirmed that experimental upregulation of EP300 led to a decrease in the percentage of ALDH^+^ CAL51 cells (from ~43% in CAL-ev to ~23% in CAL-EP300; Fig. 3d). Thus, there is an inverse relationship between EP300 expression and CSC markers.

**Fig. 4:**
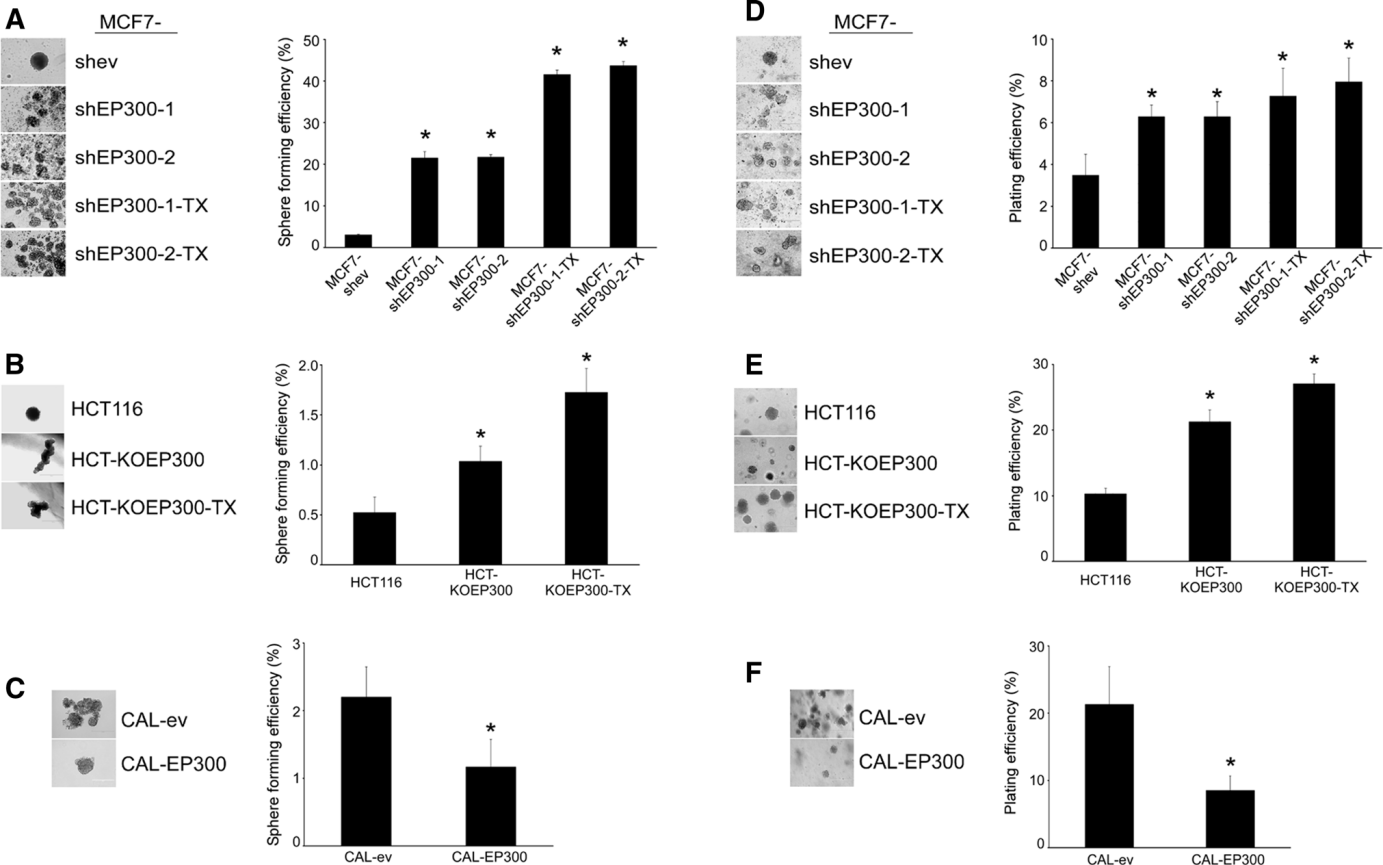
EP300 regulates anchorage independence. Anchorage independence was determined by mammosphere formation in low attachment plates (**a**–**c**) and the formation of clones in soft agar (**d**–**e**) after 2 and 4 weeks, respectively. Representative pictures of at least three independent experiments are shown. Numerical data indicate the anchorage independence efficiency after counting tumour spheres larger than 50 µm in diameter and is represented as the average ± SD of three independent experiments. All statistical comparisons (*P < 0.05) versus control cells

In order to assess the functionality of the stem cell markers above, two complementary anchorage independence assays were performed, sphere formation in liquid cultures using low attachment plates and colony formation in soft agar. Downregulation of EP300 in MCF-7 cells led to an increase in both the number of mammospheres (~7-fold) and colonies in soft agar (~2-fold). Paclitaxel-resistant derivatives showed a further two-fold increase in the number of mammospheres, although this was not observed in the formation of colonies in soft agar (Fig. 4a, d). These results were confirmed in HCT116 cells. Thus, the number of mammospheres increased two-fold and 3.5-fold in HCT-KOEP300 and HCT-KOEP300-TX cells, respectively, whereas the number of colonies in soft agar increased by two-fold and 2.5-fold in the same cells (Fig. 4b, e). Conversely, CAL51 cells overexpressing EP300 showed a 50% reduction in the anchorage-independent growth efficiency (Fig. 4c, f). MDA-MB-231, which is a cell line notoriously recalcitrant for these types of assay [28], did not generate robust tumor spheres.

Overall, these results indicate a negative regulation of CSC characteristics by EP300.

### Genome-wide expression analysis indicates EP300 has a role in regulating adhesion, apoptosis and stemness

**Fig. 5:**
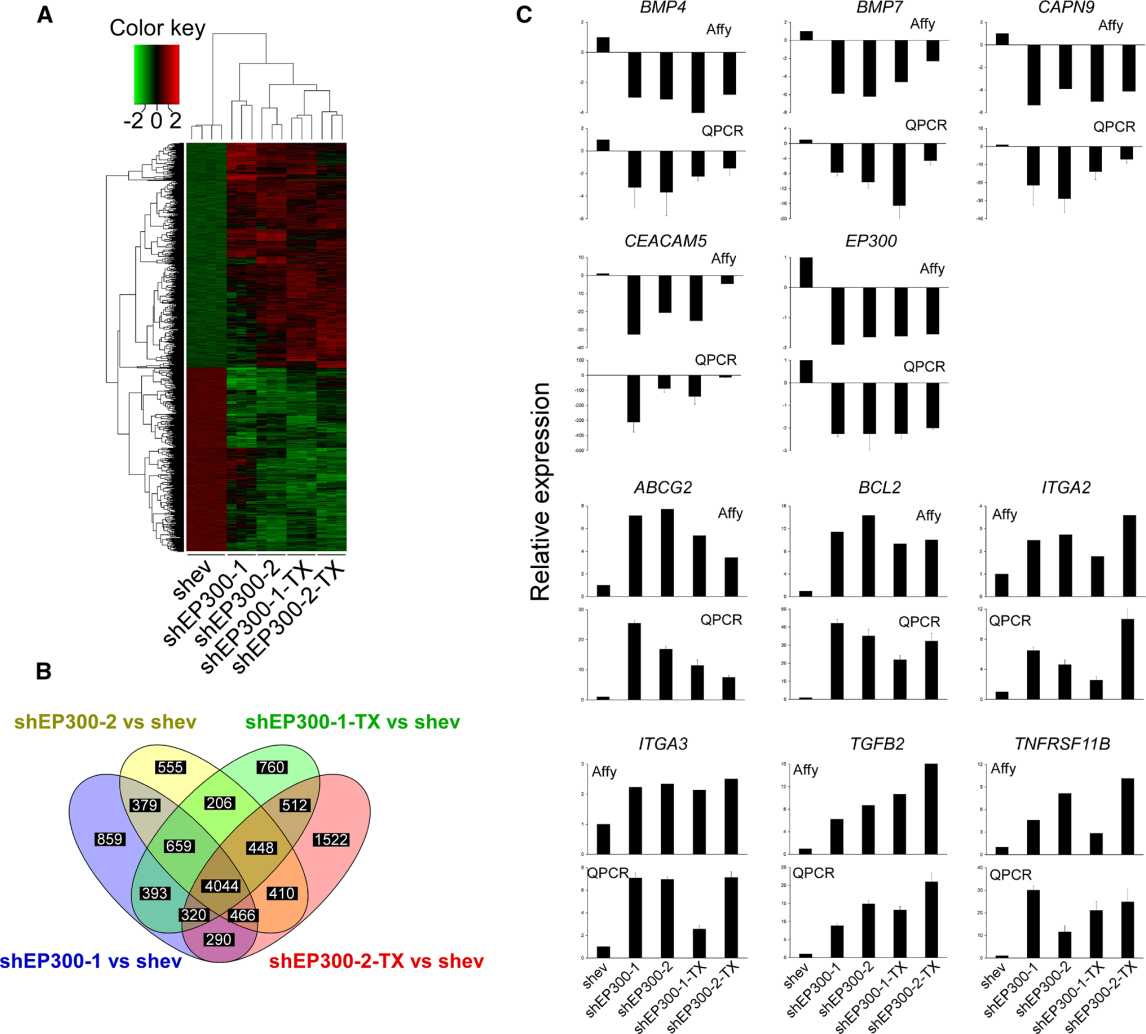
Genome-wide expression profile of EP300-downregulated MCF-7 cells, and their paclitaxel-resistant derivatives. **a** Hierarchical clustering of differentially expressed genes. Differentially upregulated expression values are shown in *red*, downregulated in *green*. *Scale* represents *colour* values corresponding to lg_2_ expression. **b**, Venn diagram indicating the number of differentially expressed genes in each of the pair-wise comparisons to MCF7-shev control cells. There were 4044 common differentially expressed genes present in all cells after downregulation of EP300. **c** Eleven genes were selected for validation by quantitative PCR. The top panel for each gene shows the normalized fluorescence from Affymetrix array expression data. The lower panel for each gene indicates the normalized QPCR data relative to the expression data obtained in control MCF7-shev cells. QPCR data represent the mean ± SD from three replicates

**Fig. 6:**
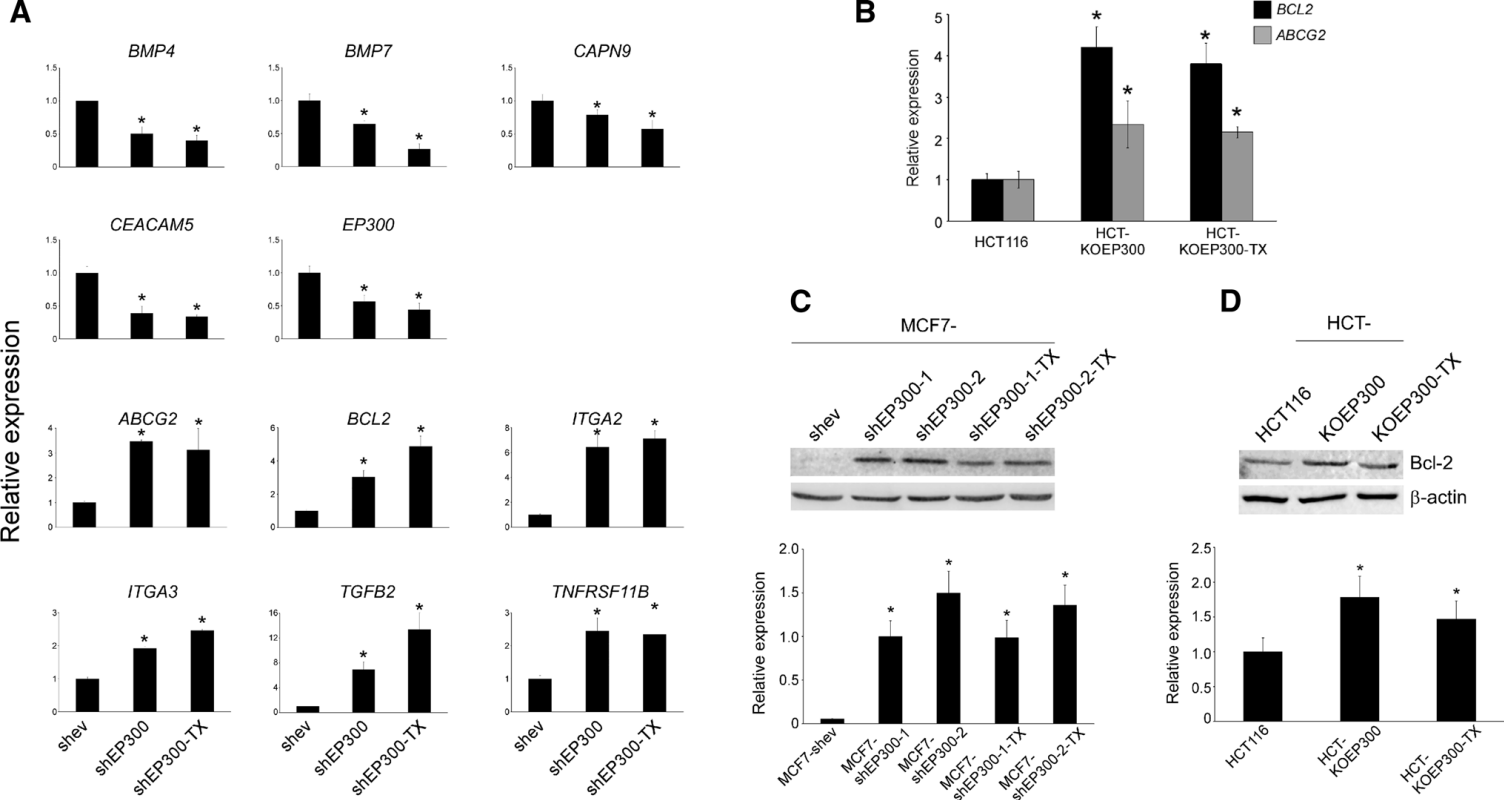
Validation of the EP300 signature in other cell models. **a** QPCR data from EP-downregulated minimally transformed mammary epithelial cells, and their paclitaxel-resistant derivatives. The gene set is the same that is used in Fig. 5c to validate the MCF-7 signature. **b** QPCR data of anti-apoptotic *BCL2* and stem cell marker *ABCG2* from the MCF-7 signature in HCT116 cells. **c** and **d**, Bcl-2 immunoblot (*top panels*) and blot quantification (*lower panels*) in both MCF-7 (**c**) and HCT116 (**d**) cell derivatives. β-actin is used as a loading control. Numerical data represent the average ± SD of three independent experiments. All statistical comparisons (*P < 0.05) versus control cells. Immunoblot shows a representative of three independent experiments

In order to gain insights into the down-stream molecules regulated by EP300, and potential effectors of the phenotypes described above, a genome-wide expression profile analysis was performed in MCF7-shEP300 cells and their paclitaxel-resistant derivatives (Fig. 5a). As expected for a chromatin modifier and regulator of transcription, more than 4000 genes were differentially regulated upon EP300 downregulation and in paclitaxel-resistant derivatives (Fig. 5b). In order to validate the array data, in addition to EP300, the expression of another 10 genes was determined by QPCR. Expression of both up- and downregulated genes was highly reproducible between the two platforms (Fig. 5c), giving a high degree of confidence for the obtained gene expression profiles. The most differentially regulated genes are shown in Supplementary Table 3. Importantly, expression of key molecules in the MCF-7 signature, such as *CEACAM5* (adhesion), *CAPN9* (cytoskeletal remodelling), *ABCG2* (stemness), *BCL2* and *TNFRSF11B* (apoptosis), *ITAG2* and *ITAG3* (migration) and *TGFB2* (metastasis), among others, was reproduced in MTMEC-shEP300 cells (Fig. 6a). Moreover, upregulation of *BCL2* and *ABCG2* was also demonstrated in both HCT-KOEP300 cells and its paclitaxel-resistant derivative (Fig. 6b, c), indicating that regulation of these two important molecules involved in apoptosis response/drug resistance and stemness is not breast cancer specific and probably represents major EP300-regulated pathways.

### EP300 is downregulated in metaplastic breast cancer

**Fig. 7:**
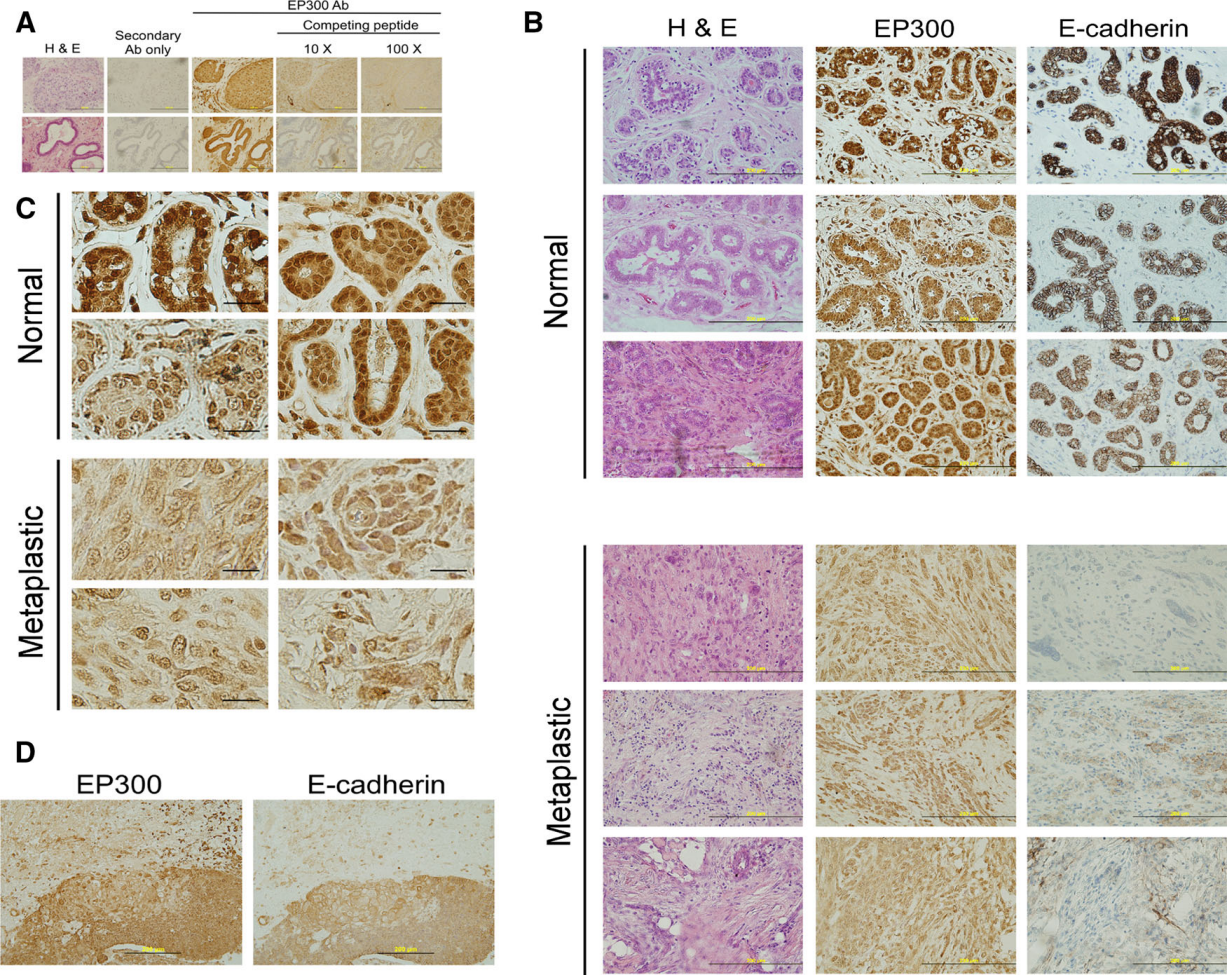
EP300 is downregulated in metaplastic breast cancer. **a** Validation of EP300 antibody using 10- and 100-fold molar excess competing peptide in two independent breast cancer samples. *H&E,* haematoxylin and eosin staining. *Scale bar*, 200 μm. **b** EP300 and E-cadherin staining in three representative samples of normal breast and metaplastic breast cancer. *H&E,* haematoxylin and eosin staining. *Scale bar*, 200 μm. **c** Loss of EP300 nuclear staining in metaplastic breast cancer. *Pictures* show representative zoomed-in shots illustrating nuclear EP300 localization in normal breast and its loss in metaplastic breast cancer. *Scale bar*, 20 μm. **d** Representative EP300 and E-cadherin staining in a metaplastic breast cancer sample with a squamous epithelium nest (bottom half). The top half is composed of spindle-like cells. Note the absence of E-cadherin and EP300 expression in the mesenchymal component but positive staining in the squamous nest. *Scale bar*, 200 μm

Metaplastic breast cancer is a rare (less than 1% of invasive breast cancers) and histologically diverse subtype of breast carcinoma with very poor prognosis. It shows the characteristics of cells undergoing EMT (spindle morphology, reprogramming of epithelial–mesenchymal markers), with acquisition of CSCs markers (CD44^+^) and is more resistant to chemotherapy than basal-like or luminal cancers [29, 30]. As EP300 regulates most of the characteristics associated with these phenotypes, we asked whether EP300 would be downregulated in metaplastic breast cancer. In order to detect EP300 by immunohistochemistry, we first validated a commercial antibody used for the Human Protein Atlas [31] by peptide competition (Fig. 7a). EP300 expression in normal/benign breast tissue was high in both the epithelial and stroma compartments. However, epithelial cells showed both nuclear and cytoplasmic staining, whereas cells from the stroma showed exclusively nuclear staining. As expected, E-cadherin expression was strong in the epithelial component and absent in the stroma (Fig. 7b). We analysed 17 metaplastic breast cancer samples that all scored negative or low for E-cadherin expression (scores 0 or 1) and found that all of them also scored low (score 1) for EP300 expression in the mesenchymal metaplastic component (Fig. 7b). Importantly, most of the EP300 nuclear staining was lost in metaplastic samples, their low positivity being the result of cytoplasmic expression (Fig. 7c). Eight (47%) metaplastic breast cancers were histologically heterogeneous, mainly with the nests of squamous differentiation showing positivity for both E-cadherin and EP300 (Fig. 7d). Due to the rarity of this type of breast cancer and the small sample size, no associations with other clinical parameters were sought. Thus, EP300 is downregulated in metaplastic breast cancer.

## Discussion

We have recently described a novel pathway controlling drug resistance in breast cancer cells. Upstream of the pathway, three miRs (miR-106b, miR-93 and miR-25) transcribed from the same cluster, target EP300, a transcriptional activator of E-cadherin. Upregulation of these miRs, found in aggressive basal-like, oestrogen receptor-negative, grade 3 breast cancers [13] and drug-resistant cell models, leads to a downregulation of EP300 and E-cadherin with initiation of an EMT [12]. However, miRs regulate the expression of many genes, and the contribution to EMT by miR targets other than EP300 cannot be ruled out. Here we describe that experimental modulation of EP300 in breast and colon cancer cell models alters paclitaxel sensitivity and the generation of resistant cells. Importantly, EP300 is also associated with stemness. Its downregulation is associated with increased in vitro tumorigenicity and cancer stem cell-like markers, whilst its ectopic expression in basal breast cancer cells partly rescues the epithelial, differentiated, paclitaxel-sensitive phenotype. Transcriptome analysis identifies key molecules associated with these phenotypes such as *CEACAM5* (adhesion), *CAPN9* (cytoskeletal remodelling), *ABCG2* (stemness), *BCL2* (apoptosis), *ITAG2* and *ITAG3* (migration) and *TGFB2* (metastasis). Lastly, we have unveiled that EP300 is downregulated in metaplastic breast cancer, a rare aggressive form of the disease with histological evidence of EMT and poor clinical outcome.

During the initiation of the metastatic cascade, cells undergo reprogramming to a less differentiated, therapy-resistant and morphologically changed phenotype [32]. As EP300 functions as a histone acetyltransferase [7], with the potential to modulate the expression of a plethora of genes, it is not surprising that its experimental downregulation in MCF-7 cells alters the expression of more than 4000 genes. Although the main function of EP300 as a transcriptional activator is well-established, such as in the case of *CDH1* (E-cadherin) transactivation [5], it may also lead to gene repression [33, 34]. Early work on MCF-7 cells expressing ribozymes specific for EP300 indicates that DNA damage-induced apoptosis is impaired in EP300-deficient cells [35]. In bladder cancer cells, experimental downregulation of EP300 leads to doxorubicin and cisplatin resistance [14, 15] and, using a minimally transformed model of mammary epithelial cells in which EP300 has been downregulated, we have recently found an impaired caspase-9 and caspase 3/7 activation following paclitaxel treatment [17]. EP300-deficient cells show multidrug resistance to a variety of structurally and functionally different drugs and γ-radiation, a phenotype that is ABC transporter independent [12, 17]. The EP300 signature presented here highlights the importance of *BCL*-*2* in the apoptotic evasion reported in EP300-deficient cells. Although a definitive mechanistic insight in the regulation of apoptosis by EP300 has not yet been established, EP300 has been described to be associated with SATB1, leading to the repression of *CYBB*, the key component of the phagocyte NADPH oxidase [36]. SATB1 has also been described to bind the *BCL*-*2* promoter where it has a negative transcriptional regulatory function [37]. Other EP300-associated factor, PCAF, accelerates apoptosis by repressing a GLI/BCL2/BAX axis in hepatocellular carcinoma [38]. It is thus tempting to speculate that these mechanisms may also play a role in breast cancer cells.

EP300-deficient cells increase their motility and invasive properties, both in colon and breast cancer cells [11], [12]. This phenotype involves major cellular reprogramming highlighted in the EP300 signature described here with downregulation of cell adhesion molecules, such as EPCAM5 and EPCAM6, and upregulation of collagens (COL12A1 and COL4A5) and mesenchymal OB-cadherin (CDH11), a characteristic EMT marker [39]. Upregulation of fibulins (EFEMP1 and HMCN1), which bind EGFR and regulate cell adhesion and migration, correlates with tumour progression and poor prognosis in ovarian cancer [40]. Upregulation of EPHA4, a receptor tyrosine kinase which binds ephrin family ligands, modulates cell morphology and promotes migration in glioma cells [41]. Consistently, high levels of EPHA4 correlate with a reduced overall survival in breast cancer patients [42]. Downregulation of ARHGAP20, a GTPase activator of Rho-type GTPases, favours Rho to be in the active GTP-bound state and promotes motility [43]. Reorganization of the actin cytoskeleton is also important for cellular remodelling, with the downregulation of plastin (PLS3) and other actin-binding proteins (CNN2), as well as upregulation of WIPF1, which intervenes in the disassembly of stress fibres in favour of filopodia formation. Importantly, high expression of WIPF1 is associated with poor prognosis in breast cancer [44]. Cytoskeletal remodelling is also regulated by CAPN9, a member of the calpain family. Low CAPN9 expression has been associated with poorer clinical outcome in breast cancer patients following endocrine therapy [45].

*TGFB2* is one of the three highly homologous isoforms of TGF-β found in humans. TGF-β is a potent inducer of EMT in mammary cells, the acquisition of CSC properties and chemotherapy resistance [46, 47]. TGF-β also induces FGFR switching, causing murine mammary epithelial cells to become sensitive to FGF2 [48]. A similar effect is observed in the EP300 signature, with upregulation of *TGFB2* and *FGFR2*. Importantly, FGF2 induces mesenchymal OB-cadherin (*CDH11*) expression [49], also observed in EP300-deficient MCF-7 cells. Importantly, metaplastic breast cancer is enriched in the markers of CSCs and EMT [50] and we show here low EP300 expression when compared to non-cancerous breast epithelium. Transcriptome profiling of metaplastic breast cancer indicates downregulation of epithelial phenotypes, remodelling of the extracellular matrix and EMT [51, 52]. Of note that the small sample analysed by Lien et al. indicates a concordance with differentially regulated genes in the MCF-7 EP300 signature, downregulation of *CDH1, CEACAM6* and *ARHGAP8,* another Rho GTPase-activating protein, as well as upregulation of *BCL2A1, EFNB1* (ephrin B1) and osteoprotegerin (*TNFRSF11B*), a soluble TRAIL decoy receptor secreted by inflammatory and invasive breast cancer cells that induces aneuploidy, cell proliferation and angiogenesis [53].

In conclusion, we demonstrate that tumour suppressor EP300 is poorly expressed in metaplastic breast cancer and is a master regulator of EMT, CSCs and drug resistance.

## Electronic supplementary material

Below is the link to the electronic supplementary material.

Supplementary material 1 (XLS 50 kb)

Supplementary material 2 (DOC 36 kb)

Supplementary material 3 (DOC 148 kb)

## Abbreviations

ALDH: Aldehyde dehydrogenase
CSC: Cancer stem cell
DMEM: Dulbecco’s modified Eagle medium
EMT: Epithelial-to-mesenchymal transition
IC_50_: Drug concentration producing 50% cell death
miR: Micro RNA
MTMEC: Minimally transformed mammary epithelial cell
QPCR: Quantitative PCR
