# Supplementary material for: Tumour suppressor EP300, a modulator of paclitaxel resistance and stemness, is downregulated in metaplastic breast cancer
Source: Breast Cancer Res Treat. 2017 Mar 24;163(3):461–74. doi: 10.1007/s10549-017-4202-z (PMC5427146; doi:10.1007/s10549-017-4202-z)
Supplement: Supplementary file 2 — Supplementary material 2 (DOC 36 kb) [file 10549_2017_4202_MOESM2_ESM.doc]

**Supplementary Table 2**. Standard operating procedure for the immunohistochemical staining of clinical samples using the Bond Polymer Refine Detection kit (Leica Biosystems)

| **A. Deparaffinization**  1. In Xylene - for 30 min  2. In Xylene - for another 30 min  3. In 100% ethanol - for 10 min  4. In 90% ethanol - for 10 min  5. In 70% ethanol - for 10 min  6. In distilled water - for 5 min |
| --- |
| **B. Antigen Retrieval**  1. Preheating the chamber of a water bath for approximately 30 min.  2. Heating (100 ºC) in the presence of citrate buffer (pH 6.0) for 30 min.  3. Cooling down to room temperature.  4. Washing in PBS (one quick wash followed by two subsequent washes, each for 5 min.). |
| **C. Antibody Treatment**  1. Preparing the antibody working solution by appropriate dilution in PBS.  2. Marking the area of the slide containing the tissue with an Immedge hydrophobic pen (Vector H-1007).  3. Adding the diluted antibody solution drop-by-drop from top of the slide to cover the area marked.  4. Incubating the slides at 4°C (in a fridge); kept in a slide tray (soaked with wet tissue paper to ensure proper hydration to avoid drying out) overnight. |
| **D. Washing**  1. A quick rinse in PBS followed by two subsequent washes, each for 5 min. |
| **E. Peroxide Block**  1. Adding 3-4% (w/v) H_2_O_2_ solution after a quick rinse with PBS.  2. Incubating at room temperature for 30 min with the lid on.  3. A quick rinse off with PBS followed by two washes, each for 5 min. |
| **F. Post-primary Treatment**  1. Applying on top of the slides the post-primary solution containing rabbit anti-mouse IgG (<10 µg/mL) in 10% (v/v) animal serum in Tris-buffered saline containing 0.09% ProClin 950.  2. Incubating at room temperature for 20 min with the lid on.  3. A quick rinse off with PBS followed by two washes, each for 5 min. |
| **G. Polymer Treatment**  1. Adding the polymer solution, containing anti-rabbit Poly-HRP-IgG (<25 µg/mL) in 10% (v/v) animal serum in Tris-buffered saline containing 0.09% ProClin 950.  2. Incubating at room temperature for 30 min. with the lid on.  3. A quick rinse off with PBS followed by two washes, each for 5 min. |
| **H. DAB Treatment**  1. Adding the premixed 3,3′-Diaminobenzidine (DAB) solution containing DAB Part A (66 mM DAB in a stabilizer solution) and DAB Part B (containing ≤0.1% v/v of H_2_O_2_ in a stabilizer solution).  2. Waiting for the color to develop (not more than 3 min) as observed under the microscope.  3. Counterstaining in hematoxylin (< 0.1%) solution followed by an acid-alcohol solution. |
| **I. Dehydration**  1. Switching on the down flow work station.  2. Dipping the slides (not more than 1-2 dip) in 50%, 70% and then in 100% alcohol.  3. Dipping the slides (not more than 1-2 dip) in 2 containers containing xylene. |
| **J. Mounting and Cover-slipping**  1. Adding 1-2 drop Pertex mounting medium (CellPath, Powys, UK) on top of a cover slip.  2. Putting the slide with the stained side down on to the cover slip. |
