# Supplementary material for: Tumour suppressor EP300, a modulator of paclitaxel resistance and stemness, is downregulated in metaplastic breast cancer
Source: Breast Cancer Res Treat. 2017 Mar 24;163(3):461–74. doi: 10.1007/s10549-017-4202-z (PMC5427146; doi:10.1007/s10549-017-4202-z)
Supplement: Supplementary file 3 [file 10549_2017_4202_MOESM3_ESM.doc]

Table 2. List of the most up-and down-regulated genes in MCF7-derivatives

Transcript ID	Gene Symbol	Gene Title	shEP300-1	shEP300-2	shEP300-1-TX	shEP300-2-TX
16744205	ARHGAP20	Rho GTPase activating protein 20	-17.1	-20.5	-23.6	-23.8
16862548	CEACAM5	carcinoembryonic antigen-related cell adhesion molecule 5	-32.7	-20.6	-25.1	-4.6
16862563	CEACAM6	carcinoembryonic antigen-related cell adhesion molecule 6 (non-specific cross reacting antigen)	-26.7	-21.6	-25.6	-8.2
17106357	PLS3	plastin 3 (T isoform)	-16.1	-15.8	-12.9	-14.2
16981631	GLRA3	glycine receptor, alpha 3	-15.6	-13.5	-14.6	-3.4
16875108	ZNF320	zinc finger protein 320	-7.2	-12.4	-14.7	-12.1
17070456	CA2	carbonic anhydrase II	-8.0	-9.3	-9.5	-7.2
17005223	ID4	inhibitor of DNA binding 4, dominant negative helix-loop-helix protein	-6.6	-9.7	-7.0	-6.1
16984287	PTGER4	prostaglandin E receptor 4 (subtype EP4)	-7.4	-5.6	-9.5	-3.3
16720601	MUC5B	mucin 5B, oligomeric mucus/gel-forming	-5.4	-6.8	-8.1	-5.2
17012304	HEY2	hairy/enhancer-of-split related with YRPW motif 2	-6.1	-4.9	-8.3	-6.1
17048190	CDK14	cyclin-dependent kinase 14	-6.3	-5.6	-8.3	-4.7
16912362	ID1	inhibitor of DNA binding 1, dominant negative helix-loop-helix protein	-8.1	-6.6	-4.4	-3.1
16824109	PLA2G10	phospholipase A2, group X	-5.7	-5.3	-5.9	-5.2
16877968	DPYSL5	dihydropyrimidinase-like 5	-5.5	-5.6	-5.0	-5.6
16856350	CNN2	calponin 2	-5.7	-5.1	-5.1	-4.1
16920585	BMP7	bone morphogenetic protein 7	-5.9	-6.2	-4.6	-2.3
16967513	AMTN	amelotin	-3.7	-5.0	-5.5	-4.8
16678651	CAPN9	calpain 9	-5.3	-3.9	-5.0	-4.1
16765542	ZNF385A	zinc finger protein 385A	-4.6	-4.3	-5.1	-4.1
16914469	SLC2A10	solute carrier family 2 (facilitated glucose transporter), member 10	-4.8	-4.3	-5.1	-3.3
16852463	RAB27B	RAB27B, member RAS oncogene family	-5.5	-4.4	-4.4	-2.2
17075973	DUSP4	dual specificity phosphatase 4	-4.1	-3.5	-5.3	-3.4
17005865	HIST1H2BM	histone cluster 1, H2bm	-2.5	-3.5	-3.5	-6.7
16814584	CACNA1H	calcium channel, voltage-dependent, T type, alpha 1H subunit	-5.5	-4.2	-3.9	-2.3
16983373	FAM105A	family with sequence similarity 105, member A	-5.5	-4.1	-3.6	-2.4
17013520	SASH1	SAM and SH3 domain containing 1	-5.3	-3.9	-4.4	-2.0
16685868	CITED4	Cbp/p300-interacting transactivator, with Glu/Asp-rich carboxy-terminal domain, 4	-2.8	-3.0	-5.1	-3.8
16880712	SLC1A4	solute carrier family 1 (glutamate/neutral amino acid transporter), member 4	5.4	3.3	2.4	2.2
17000168	CXCL14	chemokine (C-X-C motif) ligand 14	5.8	3.8	1.7	2.5
16869960	CYP4F11	cytochrome P450, family 4, subfamily F, polypeptide 11	5.3	4.6	2.9	2.6
16981099	NPY1R	neuropeptide Y receptor Y1	5.1	4.3	3.9	3.2
16958403	HEG1	HEG homolog 1 (zebrafish)	4.1	5.8	3.6	3.8
16897834	EFEMP1	EGF-containing fibulin-like extracellular matrix protein 1	3.2	5.1	6.0	4.8
16827041	CDH11	cadherin 11, type 2, OB-cadherin (osteoblast)	4.5	5.1	4.5	5.4
16675045	HMCN1	hemicentin 1	4.4	4.8	4.5	6.0
16719025	FGFR2	fibroblast growth factor receptor 2	3.3	6.3	4.6	5.7
17125282	PGM5	phosphoglucomutase 5	5.2	8.1	3.7	3.0
17061662	LAMB1	laminin, beta 1	6.8	7.3	3.1	4.3
16991010	SLC26A2	solute carrier family 26 (sulfate transporter), member 2	6.3	8.0	3.5	5.6
17018841	KCNK5	potassium channel, subfamily K, member 5	6.8	7.5	4.4	4.7
16977868	ABCG2	ATP-binding cassette, sub-family G (WHITE), member 2	7.1	7.7	5.4	3.5
17117463	PLXDC2	plexin domain containing 2	5.2	6.7	6.4	6.8
16905238	WIPF1	WAS/WASL interacting protein family, member 1	7.7	6.3	6.8	4.5
17106102	COL4A5	collagen, type IV, alpha 5	4.8	4.7	6.7	11.1
16690019	LPPR5	lipid phosphate phosphatase-related protein type 5	7.6	6.7	8.9	7.0
17010782	HTR1E	5-hydroxytryptamine (serotonin) receptor 1E	7.1	9.4	7.1	8.7
16908897	EPHA4	EPH receptor A4	8.6	10.3	6.3	7.5
16773131	SGCG	sarcoglycan, gamma (35kDa dystrophin-associated glycoprotein)	10.7	11.5	9.6	7.2
16947173	MME	membrane metallo-endopeptidase	7.3	11.3	6.3	15.1
16677556	TGFB2	transforming growth factor, beta 2	6.2	8.7	10.7	16.1
17020846	COL12A1	collagen, type XII, alpha 1	6.0	11.1	8.1	16.6
16702982	MALRD1	MAM and LDL receptor class A domain containing 1	9.6	10.0	12.4	11.6
17020258	BMP5	bone morphogenetic protein 5	20.3	5.1	16.3	2.8
16855673	BCL2	B-cell CLL/lymphoma 2	11.4	14.4	9.4	10.0
17010760	NT5E	5'-nucleotidase, ecto (CD73)	12.4	20.0	11.9	10.5
16970118	SYNPO2	synaptopodin 2	19.9	17.2	15.1	6.0
17059804	CALCR	calcitonin receptor	11.3	19.8	10.9	21.3
17120346	MALRD1	MAM and LDL receptor class A domain containing 1	20.7	23.0	24.7	21.8

Values represent expression fold difference with respect to MCF-shev cells
